# Food Choice Values and Food Literacy in a Nationwide Sample of Japanese Adults: Associations with Sex, Age, and Body Mass Index

**DOI:** 10.3390/nu14091899

**Published:** 2022-04-30

**Authors:** Kentaro Murakami, Nana Shinozaki, Xiaoyi Yuan, Ryoko Tajima, Mai Matsumoto, Shizuko Masayasu, Satoshi Sasaki

**Affiliations:** 1 Department of Social and Preventive Epidemiology, School of Public Health, University of Tokyo, Tokyo 113-0033, Japan; nana-s@m.u-tokyo.ac.jp (N.S.); stssasak@m.u-tokyo.ac.jp (S.S.); 2 Department of Nutritional Epidemiology and Shokuiku, National Institute of Biomedical Innovation, Health and Nutrition, Tokyo 162-8636, Japan; xiaoyi_yuan@nibiohn.go.jp (X.Y.); rtajima@nibiohn.go.jp (R.T.); m-matsumoto@nibiohn.go.jp (M.M.); 3 Ikurien-Naka, Ibaraki 311-0105, Japan; masayasu@ikurien.com

**Keywords:** diet, nutrition, food, value, literacy, knowledge, skill, behavior, sex, age, Japan

## Abstract

This cross-sectional study of 2231 Japanese adults described food choice values and food literacy in relation to sex, age, and body mass index. We assessed eight food choice values (accessibility, convenience, health/weight control, tradition, sensory appeal, organic, comfort, and safety, using a 25-item scale), as well as food literacy, which was characterized by nutrition knowledge (using a validated 143-item questionnaire), cooking and food skills (using 14- and 19-item scales, respectively), and eight eating behaviors (hunger, food responsiveness, emotional overeating, enjoyment of food, satiety responsiveness, emotional undereating, food fussiness, and slowness in eating, using the 35-item Adult Eating Behavior Questionnaire). Females had higher means of all the variables than males, except for food fussiness. Compared to participants aged 19–39 and/or 40–59 years, those aged 60–80 years had low means of some food choice values (accessibility, convenience, sensory appeal, and comfort), nutrition knowledge, and all the food approach behaviors (hunger, food responsiveness, emotional overeating, and enjoyment of food) and high means of other food choice values (tradition, organic, and safety) and slowness in eating. Age was inversely associated with cooking and food skills in males, whereas the opposite was observed in females. The associations with body mass index were generally weak. These findings serve as both a reference and an indication for future research.

## 1. Introduction

According to a global estimate, dietary factors are responsible for 11 million deaths and 255 million disability-adjusted life years (22% and 15% of total numbers, respectively) annually [1]. Not only because this magnitude is larger than any other risk factor, including tobacco smoking, but also because diet is modifiable, improving the quality of diet is now a global priority [2]. Unfortunately, “one-size-fits-all” dietary guidelines do not achieve the changes in dietary behavior needed to achieve healthier dietary patterns [3]. Considering the complex and varied nature of individual characteristics that are related to dietary behaviors [4], there is great interest in investigating and understanding factors that shape food choices and eating behaviors. What is intriguing in this context is the concept of food choice values, defined as factors that individuals consider when deciding which foods to purchase and/or consume [5]. On the basis of the food choice process model [6], food choice values are supposed to represent the proximal influences on food choice and eating behaviors, conveying the effects of more distal determinants, including life course factors (such as socioeconomic factors), sociocultural resources, and cognitive resources [5].

Another concept recently emerging is food literacy. Although there are a number of definitions of food literacy in the literature [7,8,9], the most widely cited definition is that developed by Vidgen and Gallegos [10]. In their definition, food literacy is described as “a collection of inter-related knowledge, skills and behaviors required to plan, manage, select, prepare and eat food to meet needs and determine intake” [10]. Thus, food literacy is not just nutrition knowledge but also includes skills and behaviors, from knowing where food comes from to the ability to select and prepare these foods and behave in ways that meet dietary guidelines [11].

Measurement and investigation of food choice values [5,12,13,14,15,16,17,18,19] and food literacy [7,8,9,10,11,20,21,22] have been almost exclusively conducted in Western countries, with no information available in Japan. Although it is widely perceived that the diet consumed by the Japanese population is healthy, recent evidence suggests that the overall diet quality in Japanese adults is far from optimal and that there are different nutritional concerns between Japan and Western countries [23,24,25]. To formulate meaningful dietary guidelines and public health messages and develop effective intervention strategies to promote healthy eating, a comprehensive report on food choice values and food literacy among the general population is imperative.

Therefore, the aim of the present cross-sectional study was to describe food choice values and food literacy in a nationwide sample of Japanese adults aged 19–80 years, with a particular focus on their associations with sex, age, and body mass index (BMI). Informed by the definition mentioned above [10], food literacy was characterized by nutrition knowledge, cooking skills, food skills, and eating behaviors.

## 2. Materials and Methods

### 2.1. Study Procedure and Participants

This cross-sectional analysis was based on a nationwide questionnaire survey conducted between October and December 2018. The target population consisted of adult participants in the MINNADE (MINistry of health, labor and welfare-sponsored NAtionwide study on Dietary intake Evaluation) study, a nationwide dietary survey, in which an 8-day weighed dietary record was collected [26]. Participants were apparently healthy Japanese adults living in private households in Japan. Inclusion criteria consisted of willingness to participate and community-dwelling (free-living) individuals. Exclusion criteria were dietitians, individuals living with a dietitian, those working with a research dietitian, those who had received dietary counseling from a doctor or dietitian, those taking insulin treatment for diabetes, those receiving dialysis treatment, and pregnant or lactating women. Participation of only one person per household was permitted. Initially, 32 (of 47) prefectures, accounting for >85% of the total population of Japan, were selected on the basis of geographical diversity and feasibility of the survey. After being recruited in person or by email, a total of 475 research dietitians agreed to support the study by collecting data. They then recruited participants from local communities. The non-random sampling procedure was performed to reflect the proportion of the overall Japanese population in each region but with the intention to recruit an equal number of males and females.

Of 2983 adult participants in the MINNADE study (*n* = 126 for age 19 years, 480 for age 20–29 years, 476 for age 30–39 years, 475 for age 40–49 years, 474 for age 50–59 years, 479 for age 60–69 years, and 473 for age ≥ 70 years), 2248 individuals participated in the present study (response rate: 75%). For analysis, we excluded participants with missing information related to the variables of interest (*n* = 5) and those aged outside the 19–80 year age range (*n* = 12), leaving 2231 participants aged 19–80 years.

The study was conducted according to the guidelines laid out in the Declaration of Helsinki, and all procedures involving human subjects were approved by the Ethics Committee of the University of Tokyo Faculty of Medicine (protocol code: 12031; date of approval: 17 July 2018). Written informed consent was obtained from each participant and from a parent or guardian for participants aged <20 years.

### 2.2. Basic Characteristics

All information was collected by questionnaires specially designed for this survey. Responses to all questions (except for those with regard to nutrition knowledge) were checked by staff at the study center. If any responses were missing, the participant was asked to complete the questions again in person or by telephone. Sex was self-reported. Age at the time of the study was calculated based on birth date of the participant and the date the questionnaires were answered. BMI (in kg/m^2^) was calculated using self-reported body height and weight.

### 2.3. Food Choice Values

Food choice values were assessed by the Japanese version of the food choice values scale. First, the original English version of the food choice values scale [5] was translated into Japanese by a member of the research team (consisting of doctoral students and postdoctoral researchers in nutritional epidemiology) with a high level of English proficiency. Second, back translation was conducted by another member of the research team. A researcher with expertise in nutritional epidemiology and eating behaviors oversaw the translation process and approved the Japanese version and its back-translated version. The backward translations were then reviewed by researchers involved in the development and validation of the original English version of the food choice values scale [5]. Furthermore, the forward and backward translations were reviewed by an independent person who is fluent in both English and Japanese. Based on their feedback, relevant modifications were made such that the translated version better reflected the original scale.

The food choice values scale is a 25-item, self-administered questionnaire measuring eight factors of food choice values: accessibility, convenience, health/weight control, tradition, sensory appeal, organic, comfort, and safety [5]. The validity of the original English version has been described elsewhere [5]. Participants were asked to answer how important each item is when deciding what foods to buy or eat on a daily basis. The possible responses, based on a Likert scale, ranged from 1 to 5 (1: not at all, 2: a little, 3: moderately, 4: quite a bit, and 5: very). The score for each factor was calculated by the sum of the scores divided by the number of items (4 items for organic and 3 items for the other factors), with possible scores ranging from 1 to 5. In the present study population, the Cronbach’s alpha for the assessment of internal consistency was 0.69 for accessibility, 0.87 for convenience, 0.82 for health/weight control, 0.69 for tradition, 0.61 for sensory appeal, 0.85 for organic, 0.74 for comfort, and 0.78 for safety, which was comparable to observations in previous studies (range: 0.54 to 0.89) [5,12].

### 2.4. Food Literacy

In this study, food literacy was characterized by nutrition knowledge, cooking and food skills, and eating behaviors. This was based on the most widely used description of food literacy: “a collection of inter-related knowledge, skills and behaviors required to plan, manage, select, prepare and eat food to meet needs and determine intake” [10].

#### 2.4.1. Nutrition Knowledge

To assess nutrition knowledge, we used the Japanese general nutrition knowledge questionnaire (JGNKQ) [27]. As details of the structure, validity, and reliability of the JGNKQ are available elsewhere [27], only a brief description is provided here. Originally, the JGNKQ was a 147-item, self-administered questionnaire consisting of 5 sections: dietary recommendations, sources of nutrients, choosing everyday foods, diet-disease relationships, and reading a food label. The JGNKQ used in this study is a 143-item version in which 4 items with a very low prevalence of correct answers in the original version were removed. For each item, the correct response was assigned 1 point, whereas an incorrect or missing response was assigned 0 point. Thus, the possible total score ranged from 0 to 143, with a higher score reflecting a higher level of nutrition knowledge. In the present study population, the Cronbach’s alpha for the 143 items was 0.96, which was comparable to that observed in the development process of the JGNKQ (0.95) [27].

#### 2.4.2. Cooking and Food Skills

Cooking skills and food skills were assessed by the Japanese version of the English scale for cooking and food skills [28]. The development process of the Japanese scale was similar to that for the food choice values scale described above; thus, the final back-translated version was reviewed by researchers involved in the development and validation of the original English version [28].

The cooking and food skills scale is a self-administered questionnaire, which consists of 14 questions on the former and 19 questions on the latter [28]. Questions on cooking skills ask about cooking methods and food preparation techniques, whereas those on food skills ask about meal planning and preparation, shopping, budgeting, resourcefulness, and label reading/consumer awareness. The validity of the original English version has been described elsewhere [28]. Based on a 7-point Likert scale, participants were asked to rate how well they felt they performed each of the skills described (1: very poor, 7: very good). An option of “never/rarely do it” was also available for participants who considered that a skill is not used; a score of zero was assigned when this response was selected. The scores of cooking skills and food skills were calculated as the sum of all the items, with possible scores ranging from 0 to 98 for the former and from 0 to 133 for the latter. In the present study population, the Cronbach’s alpha was 0.95 for the 14 cooking skill items and 0.96 for the 19 food skill items, which was higher than those observed in previous studies (range: 0.78 to 0.94) [28].

#### 2.4.3. Eating Behaviors

Eating behaviors were assessed by the Japanese version of the Adult Eating Behaviour Questionnaire (AEBQ) prepared based on the original English version [29]. The development process of the Japanese AEBQ was similar to that for the food choice values scale described above; thus, the final back-translated version was reviewed by researchers involved in the development and validation of the original English version [29].

The AEBQ is a 35-item, self-administered questionnaire, measuring 4 food approach scales, namely hunger (5 items), food responsiveness (4 items), emotional overeating (5 items), and enjoyment of food (3 items), as well as 4 food avoidance scales, namely satiety responsiveness (4 items), emotional undereating (5 items), food fussiness (5 items), and slowness in eating (4 items) [29]. The validity of the original English version has been described elsewhere [29]. Item responses were rated based on a 5-point Likert scale ranging from “strongly disagree” to “strongly agree”, and a mean score was calculated for each scale (possible score ranging from 1 to 5). In the present study population, the Cronbach’s alpha was 0.69 for hunger, 0.68 for food responsiveness, 0.86 for emotional overeating, 0.87 for enjoyment of food, 0.68 for satiety responsiveness, 0.89 for emotional undereating, 0.79 for food fussiness, and 0.65 for slowness in eating, which was, except for slowness in eating, comparable to those observed in previous studies (range: 0.67 to 0.97) [29,30,31,32,33].

### 2.5. Statistical Analysis

Data are presented as means ± standard deviations. Pearson correlation coefficients were calculated between food choice values and food literacy (characterized by nutrition knowledge, cooking and food skills, and eating behaviors). Differences in these variables between sex and across age categories (19–39, 40–59, and 60–80 years) were examined based on an independent *t*-test and analysis of variance (followed by Bonferroni’s post hoc test), respectively. Associations of food choice values and food literacy with BMI were examined using Pearson correlation coefficients. These associations were also examined using BMI categories (≤18.5, >18.5 to <25, and ≥25 kg/m^2^), but the general impressions were similar to those obtained from correlation analyses; thus, we decided to present only correlation analyses. All statistical analyses were performed using SAS statistical software (version 9.4, SAS Institute Inc., Cary, NC, USA). We considered two-tailed *p* values <0.05 statistically significant.

## 3. Results

The present analysis included 2231 Japanese adults (1068 males and 1163 females aged 19–80 years) with a mean age of 50 years (Table 1). The mean BMI (kg/m^2^) was 23.7 (standard deviation: 3.3) for males and 22.3 (standard deviation: 3.5) for females.

### 3.1. Descriptive Statistics

Table 2 shows descriptive statistics for food choice values and food literacy variables (nutrition knowledge, cooking and food skills, and eating behaviors). For food choice values (maximum score: 5), the highest mean score was observed in safety (3.32), followed by sensory apparel (3.28) and accessibility (3.19), whereas the lowest mean score was observed in tradition (2.09). The mean score of nutrition knowledge was 70.2 (maximum score: 143), whereas the mean score of cooking and food skills was 43.3 (maximum score: 98) and 62.5 (maximum score: 133), respectively. Among eating behaviors (maximum score: 5), the highest mean score was observed in enjoyment of food (4.02), whereas the lowest mean score was observed in emotional overeating (2.37).

Pearson correlation coefficients between these variables are also shown in Table 2. The eight food choice value scores were modestly and positively correlated with each other (>0.30), with a few exceptions (between accessibility and tradition and between convenience and tradition). The highest correlation was 0.81 between organic and safety scores. Nutrition knowledge was modesty and positively correlated with both cooking and food skills (0.34 and 0.36, respectively), whereas its correlations with each of the food choice values (0.11 to 0.27) and eating behaviors (−0.16 to 0.12) were generally weak.

There was a strong correlation between cooking and food skills (0.84). Cooking and food skills were, in general, modestly correlated with each of the food choice values (0.12 to 0.41). Conversely, the correlations between cooking and food skills and each of the eating behavior scores were generally weak (−0.23 to 0.19).

The correlations between eating behavior scores varied. Positive and modest correlations were observed between the food approach scales, i.e., hunger, food responsiveness, emotional overeating, and enjoyment of food (0.19 to 0.54). Positive and modest correlations were also observed between some food avoidance scales (0.30 between satiety responsiveness and emotional undereating; 0.28 between satiety responsiveness and slowness in eating). There was a modest and inverse correlation between enjoyment of food and food fussiness (−0.38). Other correlations were rather weak (−0.18 to 0.21). The correlations between each eating behavior and each food choice value were also rather weak (−0.16 to 0.26). When correlation coefficients were calculated for males (Table S1) and females (Table S2) separately, the general interpretation and conclusion were not altered materially.

### 3.2. Association with Sex and Age

**Figure 1:**
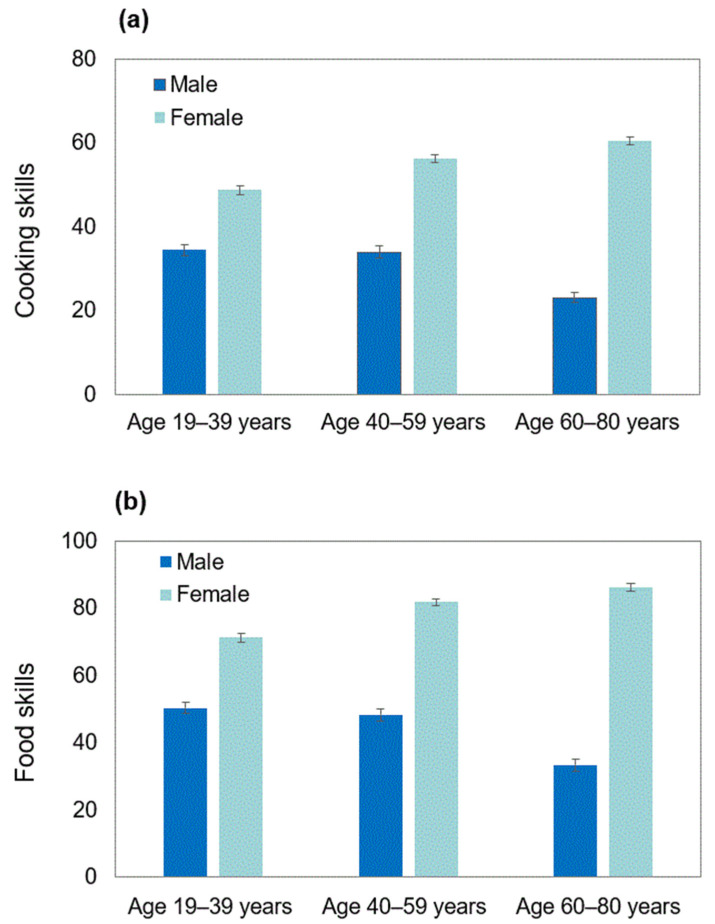
Cooking skills (**a**) and food skills (**b**) according to age category in each sex. Values are means ± standard errors. The possible score is 0 to 98 for cooking skills and 0 to 133 for food skills. The number of males is 332, 359, and 377 for age 19–39, 40–59, and 60–80 years, respectively. The number of females is 375, 392, and 396 for age 19–39, 40–59, and 60–80 years, respectively. In males, both cooking and food skills in the 60–80-year group are significantly different from the two other age groups (*p* < 0.05 by Bonferroni’s post hoc test). In females, both cooking and food skills are significantly different between three age groups (*p* < 0.05 by Bonferroni’s post hoc test).

Table 3 shows food choice values, nutrition knowledge, cooking and food skills, and eating behaviors according to sex and age categories. Compared to males, females had high mean values of all the scores examined, except for food fussiness. These sex differences were also observed when analyses were stratified by three age categories or BMI tertiles (data not shown). Age was associated with all the food choice value scores, except for health/weight control, with low scores for accessibility, convenience, sensory appeal, and comfort and high scores for tradition, organic, and safety observed in adults aged 60–80 years compared with those aged 19–39 years, those aged 40–59 years, or both. Nutrition knowledge was higher in adults aged 19–39 years and 40–59 years than in those aged 60–80 years, whereas cooking and food skills were higher in adults aged 40–59 years than in the other age groups. For eating behaviors, all the food approach scales (hunger, food responsiveness, emotional overeating, and enjoyment of food) were lower in the oldest age group. Conversely, age was not associated with the food avoidance scales (satiety responsiveness, emotional undereating, and food fussiness), except for a higher score of slowness in eating in the oldest age group. These age differences were also observed when analyses were stratified by sex or BMI tertiles (data not shown), except for food and cooking skills in sex-stratified analyses. As shown in Figure 1, both cooking and food skill scores were inversely associated with age in males, whereas these scores were positively associated with age in females.

### 3.3. Association with BMI

Table 4 shows the Pearson correlation coefficients of BMI with each of the food choice values, nutrition knowledge, cooking and food skills, and eating behaviors. In the total sample, the correlations for food choice values, nutrition knowledge, and cooking and food skills were quite weak (−0.08 to 0.11). The correlations for eating behavior scores were also not strong, ranging from −0.19 (satiety responsiveness) to 0.21 (emotional overeating). Analyses stratified by sex or age categories did not change the results materially.

## 4. Discussion

To our knowledge, this is the first comprehensive report on food choice values and food literacy (nutrition knowledge, cooking and food skills, and eating behaviors) in Japanese adults. Consistent with previous Western studies using the same [12,13] or similar [14] scales of food choice values, the three most important values were safety, sensory appeal, and accessibility. The mean score and standard deviation of nutrition knowledge in this study were astonishingly similar to those observed in a previous Japanese study (mean: 69.3; standard deviation: 23.7) [34]. Our mean estimate of cooking skills and food skills was lower and higher, respectively, than that in a nationally representative sample of the island of Ireland (47.8 and 45.8, respectively) [28]. Finally, the mean of eating behavior scores was generally similar to that reported in previous studies [29,30,31,32,33], particularly in that the highest score was obtained for enjoyment of food. These basic findings confirm the validity of the measures in the present study.

The correlations between each of the food choice values [5,12] and between each of the eating behaviors [29,30,31,32,33] were generally similar to those reported in previous studies. The strong correlation between cooking and food skills observed here is consistent with the results of previous studies [28,35]. The present study also found modest and positive associations of cooking and food skills with nutrition knowledge, which is plausible. However, as we are unaware of previous studies in this regard, further research is needed.

We found that females had higher means of food choice values [17,18], nutrition knowledge [34,36,37], and cooking and food skills [35,38], all of which is consistent with several Western studies. These findings may reflect that women still have the main responsibility for cooking and probably grocery shopping in many households in Japan [39]. A study also suggested that women tend to be exposed to stronger sociocultural norms for body shape [40]. These factors may lead to greater involvement and preoccupation with food among women [19]. For eating behaviors, sex differences have not been extensively examined using the same questionnaire. However, in a French-speaking Canadian population, emotional overeating, satiety responsiveness, and emotional undereating were higher in females than in males [32], which is consistent with the findings of the present study.

Generally, previous studies [14,19,41] have suggested that older individuals tend to value more “long-term-oriented” motives (e.g., health/weight control, organic, and safety), whereas younger individuals tend to emphasize more “short-term-oriented” motives (e.g., accessibility, convenience, and sensory appeal). We observed consistent associations. As suggested by Konttinen and colleagues, the emergence of diverse health problems and the phase of the life course (e.g., having children and more stabilized life and financial situation) are likely to contribute to more long-term and health-conscious orientation in middle-aged and older adults [19]. Nevertheless, we found no association between age and health/weight control in this study. The exact reason is unknown, but this may be due to higher health consciousness in younger individuals, lower health consciousness in older individuals, or both, as well as the general lean nature of the Japanese population. Further studies are warranted.

In this study, nutrition knowledge was higher in adults aged 19–39 years and 40–59 years than in those aged 60–80 years, although the difference was not large. The associations between nutrition knowledge and age varied in previous studies. For example, there was no significant association between nutrition knowledge and age in an analysis of Japanese adults aged 18–64 years (mean age 44 years) [34]. Conversely, nutrition knowledge was positively associated with age among Belgian women aged 18–39 years [42] and Australian adults aged 18–74 years (41% of participants aged 18–34 years) [36]. A study conducted in England showed that the youngest (18–34 years) and the oldest (≥65 years) age groups had a lower nutrition knowledge score than people in the middle years (35–44, 45–54, and 55–64 years) [37]. Considering the differences in age range of the populations between studies, these observations do not necessarily conflict with the findings of the present study.

One of the most important findings in this study is that age was inversely associated with cooking and food skills in males, whereas the opposite was observed in females, in addition to much higher scores for both skills in females than in males. Similar findings with regard to cooking skills have been reported in a series of Japanese studies [39,43]. This may be explained by the fact that older males had limited opportunities to learn cooking and food skills because home economics classes were conducted only for females until 1993 in junior high schools and 1994 in high schools in Japan [44]. Additionally, older males (and older females) may tend to persist in a belief in gender roles ideology that dictates that men should work outside the home and women should do housework at home [39]. Conversely, the narrower gap between sex in food and cooking skills observed in younger age groups may suggest more opportunities to learn cooking and food skills for younger males in schools, as well as gender role ideology gradually becoming obsolete. In any case, future studies should carefully consider these sex and age differences in cooking and food skills.

For eating behaviors, we observed that all the food approach scales (hunger, food responsiveness, emotional overeating, and enjoyment of food) were lower in the oldest age group. Similar findings were reported in a study of 197 French-speaking Canadian adults, although only food responsiveness reached statistical significance [32]. Consistent with the Canadian study [32], we observed no association of age with the food avoidance scales (except for slowness in eating). The higher score of slowness in eating observed in the oldest age group does fit well with our previous observation that time spent eating breakfast, lunch, and dinner was longer in the oldest age group (60–79 years) than younger age groups (20–39 and 40–59 years) [26].

In this study, the correlations of food choice values, nutrition knowledge, and cooking and food skills with BMI were generally weak. This is consistent with a few reports in which the association of food choice values [18], cooking and food skills [35], and nutrition knowledge [42] with BMI or weight status was, if present, not strong. A number of studies have reported associations between eating behaviors assessed by AEBQ and BMI (or weight status) [29,30,31,32,33]. The most consistent findings are a positive association for emotional overeating [29,30,32,33] and inverse associations for satiety responsiveness [29,30,31,33] and for slowness in eating [29,30,31,32,33], which is consistent with observations of the present study.

The strengths of the present study include the simultaneous focus on food choice values and food literacy (nutrition knowledge, cooking and food skills, and eating behaviors) and the use of well-established scales for these variables (particularly nutrition knowledge), as well as a large nationwide sample with almost the equal proportions for sex and age categories. However, there are also several limitations. First, although sampling was conducted so that regional differences in population proportion are reflected, the study population is not a nationally representative sample of the general Japanese population but rather volunteers. It is conceivable that the participants were more representative of health-conscious individuals. Nevertheless, an analysis conducted in the context of the MINNADE study [26], from which our participants were recruited (with a response rate of 75%), showed that the distribution of annual household income was similar to that in a national representative sample, although education level was somewhat higher. Furthermore, mean (standard deviation) values of body height, body weight, and BMI in the present participants were also similar to those in a national representative sample aged ≥20 years (males: 167.6 (7.0) cm, 67.0 (11.5) kg, and 23.8 (3.4) kg/m^2^, respectively; females: 154.1 (6.9) cm, 53.6 (9.4) kg, and 22.6 (3.7) kg/m^2^, respectively) [45]. Thus, there may be no strong reason for considering that the present participants largely differ from the general Japanese population. Nevertheless, we could not compare basic characteristics between study participants and individuals who declined to participate in this study (both are participants in MINNADE study) because the use of data obtained within the MINNADE study is not permitted by the Ministry of Health, Labour and Welfare, Japan [26]. Further research in a more representative sample is thus warranted.

Second, the present analysis could not take any socioeconomic variables into account because of a lack of information. However, in a previous study of 1165 Japanese adults aged 18–64 years, nutrition knowledge was not significantly related to education or household income [34]. It is generally acknowledged that education is a strong determinant of future employment and income, and that knowledge and skills are attained through education, affecting a person’s cognitive functioning [46]. Previous Western studies have also indicated that the associations of age and sex with food choice values [17,18], except for values related to price cheapness of food [19], as well as cooking and food skills [35,38], were stronger than those for education, whereas nutrition knowledge was strongly associated with education [36,37,42]. Taken together, it is unlikely that socioeconomic factors entirely explain the findings observed here. Nevertheless, future research should incorporate the assessment of socioeconomic variables to obtain more comprehensive pictures.

Third, we calculated BMI based on self-reported body weight and height, which might be biased. However, previous studies have shown that BMI calculated from self-reported weight and height is highly correlated with BMI calculated from measured values [47,48]. It is thus considered that BMI calculated from self-reported weight and height is a reliable measure, at least for use in correlation analysis.

Finally, during the development process of the Japanese versions of assessment tools for food choice values, cooking and food skills, and eating behaviors, we did not take into account cultural differences between Japan and Western countries. This was because our main intention was to maximize the comparability of our Japanese versions with the original English versions. Consequently, the tools may not be optimal for use in the Japanese population. However, it should be noted that the internal consistency of all the scores, except for slowness in eating, was comparable to that observed in previous studies, as mentioned above. Additionally, the associations observed here are not only plausible but also generally comparable with previous Western studies, which confirms, to a certain extent, the validity of the measures in the present study. Nevertheless, future refinement or modification of the assessment tools specially designed for the Japanese population would be of interest.

In conclusion, we provided comprehensive pictures of food choice values (accessibility, convenience, health/weight control, tradition, sensory appeal, organic, comfort, and safety) and food literacy, which was characterized by nutrition knowledge, cooking and food skills, and eating behaviors (hunger, food responsiveness, emotional overeating, enjoyment of food, satiety responsiveness, emotional undereating, food fussiness, and slowness in eating) in a large nationwide sample of Japanese adults aged 19–80 years. The major findings are as follows: compared to males, females had high means of all the variables, except for food fussiness; compared to participants aged 19–39 and/or 40–59 years, those aged 60–80 years had low means of some food choice values (accessibility, convenience, sensory appeal, and comfort), nutrition knowledge, and all the food approach behaviors (hunger, food responsiveness, emotional overeating, and enjoyment of food), as well as high means of other food choice values (tradition, organic, and safety) and slowness in eating; age was inversely associated with cooking and food skills in males, whereas the opposite was observed in females; the associations with BMI were generally weak. These observations in Japanese adults are generally consistent with those in Western countries, which is interesting, considering the widespread perception of the Japanese diet as healthful; further research is warranted. The present findings serve as both a reference and an indication for future research on food choice values and food literacy in Japan.

## Figures and Tables

**Table 1:** Basic characteristics of the study population ^1^.

Variable	All (*n* = 2231)	Male (*n* = 1068)	Female (*n* = 1163)
Age (years)	50.2 ± 17.3	50.4 ± 17.2	50.0 ± 17.5
Body height (cm) ^2^	162.6 ± 8.9	169.4 ± 6.3	156.3 ± 5.9
Body weight (kg) ^2^	60.9 ± 12.1	68.0 ± 10.9	54.4 ± 9.0
Body mass index (kg/m^2^) ^3^	22.9 ± 3.5	23.7 ± 3.3	22.3 ± 3.5

^1^ Values are means ± standard deviations. ^2^ Based on self-report. ^3^ Calculated using self-reported body height and weight.

**Table 2:** Descriptive statistics of food choice values and food literacy variables characterized by nutrition knowledge, cooking and food skills, and eating behaviors (*n* = 2231) ^1^.

		Pearson Correlation Coefficient																		
Variable	Mean ± SD	1	2	3	4	5	6	7	8	9	10	11	12	13	14	15	16	17	18	19
Food choice values																				
Accessibility (1)	3.19 ± 0.78	---																		
Convenience (2)	3.09 ± 0.85	0.55	---																	
Health/weight control (3)	2.82 ± 0.91	0.33	0.39	---																
Tradition (4)	2.09 ± 0.75	0.25	0.18	0.35	---															
Sensory appeal (5)	3.28 ± 0.70	0.48	0.40	0.37	0.32	---														
Organic (6)	2.95 ± 0.84	0.37	0.33	0.54	0.50	0.40	---													
Comfort (7)	2.33 ± 0.82	0.34	0.35	0.46	0.53	0.34	0.43	---												
Safety (8)	3.32 ± 0.90	0.40	0.36	0.49	0.40	0.43	0.81	0.39	---											
Nutrition knowledge (9)	70.2 ± 24.6	0.18	0.17	0.20	0.11	0.21	0.27	0.17	0.24	---										
Cooking and food skills																				
Cooking skills (10)	43.3 ± 26.0	0.20	0.12	0.20	0.22	0.23	0.34	0.19	0.25	0.34	---									
Food skills (11)	62.5 ± 34.6	0.28	0.21	0.28	0.24	0.24	0.41	0.24	0.31	0.36	0.84	---								
Eating behaviors																				
Hunger (12)	2.77 ± 0.70	0.14	0.18	0.12	0.06	0.13	0.06	0.21	0.06	0.08	0.06	0.08	---							
Food responsiveness (13)	2.74 ± 0.67	0.15	0.16	0.14	0.11	0.17	0.06	0.26	0.06	0.11	0.15	0.17	0.54	---						
Emotional overeating (14)	2.37 ± 0.79	0.06	0.10	0.17	0.12	0.05	0.04	0.25	0.01	0.07	0.07	0.10	0.37	0.43	---					
Enjoyment of food (15)	4.02 ± 0.74	0.12	0.09	0.15	0.08	0.21	0.13	0.17	0.11	0.12	0.18	0.19	0.28	0.46	0.19	---				
Satiety responsiveness (16)	2.59 ± 0.70	0.05	0.08	0.06	0.08	0.07	0.10	0.09	0.07	0.02	0.09	0.08	0.09	−0.01	−0.04	−0.18	---			
Emotional undereating (17)	2.73 ± 0.86	0.07	0.09	0.09	0.10	0.11	0.13	0.14	0.12	0.06	0.10	0.13	0.21	0.16	0.08	−0.01	0.30	---		
Food fussiness (18)	2.59 ± 0.78	−0.05	−0.02	−0.12	−0.07	−0.10	−0.19	−0.08	−0.16	−0.16	−0.23	−0.23	−0.06	−0.23	0.02	−0.38	0.16	0.04	---	
Slowness in eating (19)	2.57 ± 0.72	0.03	0.03	0.01	0.07	0.08	0.17	0.04	0.14	0.07	0.08	0.08	0.05	0.01	−0.06	0.02	0.28	0.15	−0.05	---

SD, standard deviation. ^1^ Possible scores range from 1 to 5 for each of the food choice values and eating behaviors, from 0 to 143 for nutrition knowledge, from 0 to 98 for cooking skills, and from 0 to 133 for food skills. In this sample size (*n* = 2231), Pearson correlation coefficients are statistically significant when values are greater than 0.0823 or less than −0.0823 at the level of *p* < 0.0001 (marked in yellow), greater than 0.0696 or less than −0.0696 at the level of *p* < 0.001 (marked in pink), greater than 0.0545 or less than −0.0545 at the level of *p* < 0.01 (marked in blue), and greater than 0.0415 or less than −0.0415 at the level of *p* < 0.05 (marked in orange).

**Table 3:** Food choice values and food literacy variables characterized by nutrition knowledge, cooking and food skills, and eating behaviors according to sex and age categories (*n* = 2231) ^1^.

Variable	Male (*n* = 1068)	Female (*n* = 1163)	*p* Value for Sex (*t*-Test)	Age 19–39 Years (*n* = 707)	Age 40–59 Years (*n* = 751)	Age 60–80 Years (*n* = 773)	*p* Value for Age Categories (ANOVA) ^2^
Food choice values							
Accessibility	3.06 ± 0.84	3.32 ± 0.71	<0.0001	3.34 ± 0.75 ^a^	3.18 ± 0.78 ^b^	3.07 ± 0.80 ^c^	<0.0001
Convenience	2.94 ± 0.91	3.23 ± 0.76	<0.0001	3.29 ± 0.82 ^a^	3.09 ± 0.85 ^b^	2.89 ± 0.83 ^c^	<0.0001
Health/weight control	2.67 ± 0.95	2.97 ± 0.84	<0.0001	2.84 ± 0.94	2.83 ± 0.88	2.80 ± 0.90	0.60
Tradition	1.98 ± 0.75	2.19 ± 0.74	<0.0001	1.94 ± 0.73 ^a^	2.09 ± 0.75 ^b^	2.23 ± 0.76 ^c^	<0.0001
Sensory appeal	3.17 ± 0.73	3.38 ± 0.65	<0.0001	3.39 ± 0.68 ^a^	3.30 ± 0.68 ^b^	3.16 ± 0.71 ^c^	<0.0001
Organic	2.72 ± 0.87	3.16 ± 0.76	<0.0001	2.73 ± 0.77 ^a^	2.95 ± 0.82 ^b^	3.15 ± 0.88 ^c^	<0.0001
Comfort	2.19 ± 0.82	2.45 ± 0.79	<0.0001	2.40 ± 0.84 ^a^	2.36 ± 0.81 ^a^	2.23 ± 0.79 ^b^	<0.0001
Safety	3.14 ± 0.95	3.48 ± 0.82	<0.0001	3.15 ± 0.89 ^a^	3.31 ± 0.88 ^b^	3.47 ± 0.90 ^c^	<0.0001
Nutrition knowledge	63.9 ± 25.8	76.0 ± 21.8	<0.0001	72.4 ± 23.2 ^a^	71.3 ± 25.0 ^a^	67.2 ± 25.2 ^b^	0.0001
Cooking and food skills							
Cooking skills	30.3 ± 25.9	55.2 ± 19.5	<0.0001	42.1 ± 23.3 ^a^	45.5 ± 25.7 ^b^	42.2 ± 28.3 ^a^	0.02
Food skills	43.6 ± 34.1	79.8 ± 24.4	<0.0001	61.4 ± 30.6 ^a^	65.7 ± 32.8 ^b^	60.3 ± 39.2 ^a^	0.005
Eating behaviors							
Hunger	2.67 ± 0.66	2.87 ± 0.72	<0.0001	2.92 ± 0.71 ^a^	2.85 ± 0.68 ^a^	2.57 ± 0.66 ^b^	<0.0001
Food responsiveness	2.58 ± 0.64	2.88 ± 0.67	<0.0001	2.93 ± 0.72 ^a^	2.76 ± 0.64 ^b^	2.53 ± 0.60 ^c^	<0.0001
Emotional overeating	2.24 ± 0.77	2.48 ± 0.80	<0.0001	2.48 ± 0.87 ^a^	2.42 ± 0.80 ^a^	2.22 ± 0.69 ^b^	<0.0001
Enjoyment of food	3.94 ± 0.76	4.09 ± 0.72	<0.0001	4.14 ± 0.77 ^a^	4.01 ± 0.72 ^b^	3.90 ± 0.71 ^c^	<0.0001
Satiety responsiveness	2.46 ± 0.66	2.72 ± 0.72	<0.0001	2.59 ± 0.74	2.60 ± 0.69	2.59 ± 0.68	0.89
Emotional undereating	2.56 ± 0.90	2.87 ± 0.80	<0.0001	2.67 ± 0.87	2.76 ± 0.86	2.74 ± 0.86	0.14
Food fussiness	2.63 ± 0.78	2.54 ± 0.77	0.0006	2.60 ± 0.79	2.55 ± 0.79	2.61 ± 0.74	0.21
Slowness in eating	2.44 ± 0.72	2.69 ± 0.70	<0.0001	2.55 ± 0.77 ^a^	2.48 ± 0.68 ^a^	2.69 ± 0.69 ^b^	<0.0001

ANOVA, analysis of variance. ^1^ Values are means ± standard deviations. Possible scores range from 1 to 5 for each of the food choice values and eating behaviors, from 0 to 143 for nutrition knowledge, from 0 to 98 for cooking skills, and from 0 to 133 for food skills. ^2^ When the overall *p* from ANOVA was <0.05, Bonferroni’s post hoc test was performed; mean values within a row with unlike superscript letters are significantly different (*p* < 0.05).

**Table 4:** Pearson correlation coefficients of body mass index with each of the food choice values and food literacy variables characterized by nutrition knowledge, cooking and food skills, and eating behaviors ^1^.

Variable	All (*n* = 2231)	Male (*n* = 1068)	Female (*n* = 1163)	Age 19–39 Years (*n* = 707)	Age 40–59 Years (*n* = 751)	Age 60–80 Years (*n* = 773)
Food choice values						
Accessibility	−0.08 ***	−0.07 *	−0.01	−0.06	−0.05	−0.08 *
Convenience	−0.05 *	−0.02	−0.02	−0.03	−0.04	−0.04
Health/weight control	0.11 ****	0.15 ****	0.16 ****	0.12 **	0.08 *	0.15 ****
Tradition	0.04	0.04	0.09 **	0.05	0.01	0.03
Sensory appeal	−0.04 *	0.00	−0.03	−0.01	−0.02	−0.07
Organic	−0.07 ***	−0.03	−0.01	−0.07	−0.11 **	−0.10 **
Comfort	0.01	0.04	0.05	−0.01	−0.01	0.07 *
Safety	−0.07 **	−0.02	−0.04	−0.05	−0.09 *	−0.11 **
Nutrition knowledge	−0.02	0.06 *	−0.01	0.06	−0.07	−0.03
Cooking and food skills						
Cooking skills	−0.05 *	0.07 *	0.05	0.03	−0.09 **	−0.08 *
Food skills	−0.08 ***	0.07 *	0.00	−0.03	−0.11 **	−0.09 *
Eating behaviors						
Hunger	−0.03	0.10 ***	−0.09 **	−0.08 *	−0.02	0.05
Food responsiveness	0.06 **	0.16 ****	0.06 *	0.04	0.15 ****	0.06
Emotional overeating	0.21 ****	0.25 ****	0.26 ****	0.20 ****	0.26 ****	0.21 ****
Enjoyment of food	0.08 ***	0.15 ****	0.06	0.03	0.12 ***	0.12 ***
Satiety responsiveness	−0.19 ****	−0.12 ****	−0.18 ****	−0.23 ****	−0.17 ****	−0.16 ****
Emotional undereating	−0.08 ***	0.01	−0.09 **	−0.11 **	−0.10 **	−0.03
Food fussiness	−0.02	−0.06 *	−0.02	−0.01	−0.05	−0.01
Slowness in eating	−0.18 ****	−0.17 ****	−0.12 ****	−0.23 ****	−0.16 ****	−0.15 ****

^1^ Body mass index (kg/m^2^) was calculated as self-reported weight (kg) divided by the square of self-reported height (m). * *p* < 0.05; ** *p* < 0.01; *** *p* < 0.001; **** *p* < 0.0001.

## Data Availability

The datasets generated and analyzed during the present study are not publicly available due to privacy and ethical restrictions imposed by the Ethics Committee of the University of Tokyo, Faculty of Medicine, but are available from the corresponding author upon reasonable request. The questionnaires used in this study are available from the corresponding author upon reasonable request.

## Supplementary Materials

The following supporting information can be downloaded at: https://www.mdpi.com/article/10.3390/nu14091899/s1, Table S1: Pearson correlation coefficients between food choice values and food literacy variables characterized by nutrition knowledge, cooking and food skills, and eating behaviors in 1068 males, Table S2: Pearson correlation coefficients between food choice values and food literacy variables characterized by nutrition knowledge, cooking and food skills, and eating behaviors in 1163 females.
