# Supplementary material for: Food Choice Values and Food Literacy in a Nationwide Sample of Japanese Adults: Associations with Sex, Age, and Body Mass Index
Source: Nutrients. 2022 Apr 30;14(9):1899. doi: 10.3390/nu14091899 (PMC9102665; doi:10.3390/nu14091899)
Supplement: Supplementary file 1 [file nutrients-14-01899-s001.zip › nutrients-1636308-supplementary.pdf]

Table S1. Pearson correlation coefficients between food choice values and food literacy variables characterized by nutrition knowledge, cooking and food skills, and eating behaviors in 1068 males.

**Variable**	**1**	**2**	**3**	**4**	**5**	**6**	**7**	**8**	**9**	**10**	**11**	**12**	**13**	**14**	**15**	**16**	**17**	**18**	**19**
Food choice values																			
Accessibility (1)	1.00																		
Convenience (2)	0.62	1.00																	
Health/weight control (3)	0.34	0.41	1.00																
Tradition (4)	0.26	0.22	0.37	1.00															
Sensory appeal (5)	0.48	0.40	0.38	0.34	1.00														
Organic (6)	0.40	0.39	0.60	0.48	0.42	1.00													
Comfort (7)	0.35	0.38	0.46	0.51	0.35	0.45	1.00												
Safety (8)	0.44	0.41	0.55	0.40	0.45	0.82	0.42	1.00											
Nutrition knowledge (9)	0.18	0.20	0.19	0.09	0.23	0.26	0.18	0.25	1.00										
Cooking and food skills																			
Cooking skills (10)	0.18	0.14	0.13	0.17	0.21	0.20	0.18	0.14	0.28	1.00	0.83								
Food skills (11)	0.26	0.25	0.23	0.19	0.24	0.29	0.22	0.22	0.31	0.83	1.00								
Eating behaviors																			
Hunger (12)	0.10	0.15	0.12	0.07	0.10	0.07	0.22	0.08	0.06	0.02	0.05	1.00							
Food responsiveness (13)	0.13	0.12	0.14	0.13	0.19	0.06	0.24	0.10	0.11	0.11	0.15	0.50	1.00						
Emotional overeating (14)	0.03	0.06	0.13	0.12	0.04	0.04	0.24	0.02	0.05	0.04	0.08	0.39	0.39	1.00					
Enjoyment of food (15)	0.14	0.10	0.14	0.10	0.25	0.15	0.17	0.14	0.10	0.15	0.19	0.20	0.42	0.16	1.00				
Satiety responsiveness (16)	0.05	0.05	0.03	0.07	0.03	0.06	0.11	0.06	−0.04	−0.03	−0.04	0.09	0.00	0.02	−0.18	1.00			
Emotional undereating (17)	0.05	0.07	0.08	0.11	0.08	0.13	0.14	0.13	0.06	0.01	0.04	0.26	0.20	0.23	0.00	0.27	1.00		
Food fussiness (18)	−0.07	−0.05	−0.14	−0.06	−0.16	−0.21	−0.09	−0.18	−0.16	−0.24	−0.24	−0.05	−0.24	0.03	−0.39	0.15	0.05	1.00	
Slowness in eating (19)	−0.01	0.00	0.01	0.04	0.04	0.18	0.02	0.14	0.01	−0.07	−0.06	0.05	−0.04	−0.06	−0.01	0.28	0.12	−0.06	1.00

In this sample size (n = 1068), Pearson correlation coefficients are statistically significant when values are greater than 0.1187 or less than −0.1187 at the level of p <0.0001, greater than 0.1005 or less than −0.1005 at the level of p <0.001, greater than 0.0788 or less than −0.0788 at the level of p <0.01, and greater than 0.0600 or less than −0.0600 at the level of p <0.05.

Table S2. Pearson correlation coefficients between food choice values and food literacy variables characterized by nutrition knowledge, cooking and food skills, and eating behaviors in 1163 females.

Variable	1	2	3	4	5	6	7	8	9	10	11	12	13	14	15	16	17	18	19
Food choice values																			
Accessibility (1)	1.00																		
Convenience (2)	0.44	1.00																	
Health/weight control (3)	0.27	0.32	1.00																
Tradition (4)	0.21	0.08	0.30	1.00															
Sensory appeal (5)	0.45	0.37	0.33	0.26	1.00														
Organic (6)	0.26	0.19	0.44	0.49	0.33	1.00													
Comfort (7)	0.29	0.26	0.42	0.52	0.31	0.36	1.00												
Safety (8)	0.31	0.25	0.39	0.38	0.38	0.78	0.32	1.00											
Nutrition knowledge (9)	0.09	0.04	0.14	0.07	0.13	0.17	0.10	0.15	1.00										
Cooking and food skills																			
Cooking skills (10)	0.07	−0.11	0.15	0.20	0.15	0.32	0.07	0.24	0.23	1.00									
Food skills (11)	0.17	−0.02	0.23	0.23	0.14	0.38	0.14	0.28	0.23	0.72	1.00								
Eating behaviors																			
Hunger (12)	0.13	0.17	0.08	0.01	0.12	−0.01	0.16	−0.01	0.04	−0.05	−0.03	1.00							
Food responsiveness (13)	0.10	0.13	0.09	0.03	0.09	−0.07	0.23	−0.06	0.01	−0.02	−0.05	0.54	1.00						
Emotional overeating (14)	0.04	0.10	0.17	0.10	0.01	−0.04	0.22	−0.07	0.01	−0.05	−0.05	0.32	0.43	1.00					
Enjoyment of food (15)	0.06	0.05	0.13	0.03	0.15	0.06	0.14	0.04	0.10	0.15	0.12	0.33	0.48	0.20	1.00				
Satiety responsiveness (16)	−0.01	0.05	0.02	0.04	0.05	0.05	0.02	0.02	0.00	0.03	0.00	0.05	−0.10	−0.15	−0.22	1.00			
Emotional undereating (17)	0.02	0.05	0.04	0.05	0.10	0.04	0.09	0.04	−0.03	0.04	0.04	0.13	0.04	−0.13	−0.06	0.29	1.00		
Food fussiness (18)	−0.01	0.03	−0.09	−0.06	−0.03	−0.16	−0.06	−0.13	−0.14	−0.22	−0.23	−0.06	−0.20	0.02	−0.37	0.19	0.05	1.00	
Slowness in eating (19)	0.01	−0.01	−0.04	0.06	0.07	0.08	0.01	0.07	0.04	0.06	0.04	0.00	−0.03	−0.13	0.01	0.24	0.12	−0.03	1.00

In this sample size (n = 1163), Pearson correlation coefficients are statistically significant when values are greater than 0.1138 or less than −0.1138 at the level of $p < 0.0001$, greater than 0.0964 or less than −0.0964 at the level of $p < 0.001$, greater than 0.0755 or less than −0.0755 at the level of $p < 0.01$, and greater than 0.0575 or less than −0.0575 at the level of $p < 0.05$.
